# Indian Multidisciplinary Expert Opinions on the Use of 20-Valent Pneumococcal Conjugate Vaccine in Adults

**DOI:** 10.3390/vaccines14100905

**Published:** 2026-10-16

**Authors:** Parvaiz A. Koul, Raja Dhar, Veeraraghavan Balaji, Vijai Kumar Ratnavelu, Randeep Guleria, Dinesh Khullar, Manisha Sahay, V. Ramasubramanian, Sanjay Kalra, Rajesh Venkitakrishnan, Agam Vora, Mangesh Tiwaskar, Mohan Bajaranglal Agarwal, Kumar Prabhash, Abraham Oomman, Madhu Gupta, Sujoy Ghosh, Raj Kumar Sharma, Rushi Deshpande, Suneetha Narreddy, Chandrashekara Srikantiah, Balebail Gopalakrishna Dharmanand, Debmalya Sanyal, Jayaraj Puthanmadathil, Sanjay Agarwal, Sachin Choudhari, Santosh Taur, Namrata Kulkarni

**Affiliations:** 1 Department of Pulmonary Medicine, Sher-I-Kashmir Institute of Medical Sciences, Srinagar 190011, India; parvaizk@gmail.com; 2 Department of Pulmonology, CK Birla Hospital, Kolkata 700027, India; 3 Department of Clinical Microbiology, Christian Medical College, Vellore 632004, India; 4 Division of Pulmonary Medicine & Critical Care, Yashoda Super Specialty Hospital, Hyderabad 500003, India; 5 Institute of Medicine & Respiratory and Sleep Medicine, Medanta Hospital, Gurugram 122001, India; 6 Department of Nephrology & Renal Transplant Medicine, Max Super Specialty Hospital, Saket Complex, New Delhi 110017, India; 7 Department of Nephrology, Osmania General Hospital, Hyderabad 500012, India; 8 Department of Infectious Diseases, Capstone Multispeciality Clinic & Apollo Hospital, Chennai 600017, India; 9 Department of Endocrinology, Bharti Hospital, Karnal 132001, India; 10 Department of Pulmonology, Rajagiri Hospital, Cochin 683112, India; 11 Department of Pulmonology, Brahma Kumari’s Global Hospital & Research Centre, Mumbai 400058, India; 12 Department of Diabetology, Shilpa Medical Research Centre, Mumbai 400068, India; 13 Department of Hematology, Bombay Hospital Institute of Medical Sciences, Mumbai 400020, India; 14 Department of Medical Oncology, Tata Memorial Hospital, Mumbai 400012, India; 15 Department of Cardiology, Apollo Hospitals, Chennai 600006, India; 16 Department of Community Medicine, Postgraduate Institute of Medical Education and Research (PGIMER), Chandigarh 160012, India; 17 Department of Endocrinology, Institute of Post Graduate Medical Education & Research, Kolkata 700071, India; 18 Department of Nephrology & Kidney Transplant Medicine, Medanta Kidney & Urology Institute, Lucknow 226030, India; 19 Department of Nephrology & Renal Transplantation, Jaslok Hospital and Research Centre, Mumbai 400026, India; 20 Department of Infectious Diseases, Apollo Health City, Hyderabad 500033, India; 21 Department of Rheumatology, ChanRe Rheumatology & Immunology Centre & Research, Bengaluru 560010, India; 22 Department of Rheumatology, Rheumacare, Bengaluru 560052, India; 23 Department of Endocrinology, KPC Medical College, Kolkata 700032, India; 24 Department of Nephrology, Mother Hospital, Thrissur 680012, India; 25 Department of Diabetes, Obesity & Metabolic Diseases, Ruby Hall Clinic & Sahyadri-Manipal Hospitals, Pune 411001, India; 26 Department of Vaccines, Pfizer Limited, Mumbai 400051, India; 27 Department of Vaccines & Digital, Pfizer Limited, Mumbai 400051, India

**Keywords:** 13-valent pneumococcal polysaccharide conjugate vaccine, 20-valent pneumococcal polysaccharide conjugate vaccine, pneumococcal infections, pneumococcal vaccines, pneumococcal disease

## Abstract

**Background:** Pneumococcal disease imposes a substantial public health burden in India, particularly among older adults and individuals with chronic or immunocompromising conditions. Although the 13-valent pneumococcal conjugate vaccine (PCV13) and the 23-valent pneumococcal polysaccharide vaccine are available, the emergence of non-PCV13 serotypes has highlighted the need for broader serotype coverage. The approval of the 20-valent pneumococcal conjugate vaccine (PCV20) offers expanded serotype coverage and the potential to simplify adult vaccination strategies. **Methods:** A multidisciplinary expert panel convened to develop evidence-informed expert opinions on PCV20 use in adults. A narrative literature review (2010–2025), a structured pre-meeting web-based survey, and structured discussions using the Nominal Group Technique were conducted. Expert opinions were refined, reviewed, and approved by all panel members. **Results:** Based on the available evidence and expert deliberations, the panel supported single-dose PCV20 for adults aged ≥50 years and at-risk individuals aged 18–49 years. The panel also provided specialty-specific recommendations to facilitate the implementation of PCV20 in routine clinical practice across diverse adult populations in India. **Conclusions:** These multidisciplinary expert opinions provide practical guidance for PCV20 use in Indian adults and are intended to complement, not replace, the formal national recommendations and to support informed clinical decision-making for adult pneumococcal vaccination in India, while acknowledging remaining uncertainty regarding effectiveness, implementation, affordability, and the need for further India-specific evidence.

## 1. Introduction

Pneumococcal disease, caused by *Streptococcus pneumoniae* (*S. pneumoniae*), is considered a significant public health burden. In 2021, the global incidence of lower respiratory tract infections due to *S. pneumoniae* was estimated at 97.9 million cases, with 505,000 associated deaths [1]. In India, pneumococcal disease accounts for approximately 4–5 million cases annually [2]. Pneumonia represents 39% of invasive pneumococcal disease (IPD), and approximately 5–11 per 1000 people have community-acquired pneumonia (CAP) [3,4].

The known risk factors for pneumococcal infections include, but are not limited to, age >50 years, preexisting heart, lung, or kidney disease and smoking [5]. Compared with healthy individuals, people with chronic heart disease have a 1.5–4 times higher risk of pneumococcal pneumonia and a 3–7 times higher risk of IPD [6]. Additionally, individuals with malignancies on immunosuppressive therapy have an increased susceptibility to IPD [7]. Despite recommendations from international and national guidelines, adult pneumococcal vaccine coverage in India remains low, contributing to a persistently high disease burden [8].

Traditionally, for adults, the 13-valent pneumococcal conjugate vaccine (PCV13) and the 23-valent pneumococcal polysaccharide vaccine (PPSV23) were available in India. The Indian consensus guideline on adult immunization [9] recommends a single dose of PCV13 followed by PPSV23 two months to one year later, and a second dose of PPSV23 after five years [9]. The increasing contribution of non-PCV13 serotypes to pneumococcal disease necessitated the subsequent use of PPSV23 to broaden serotype coverage and target clinically relevant serotypes, resulting in complex sequential vaccination schedules [10,11,12]. To address this challenge and simplify the vaccination schedules, the 20-valent pneumococcal conjugate vaccine (PCV20) has been introduced recently. This safe and well-tolerated vaccine is developed on a platform similar to PCV13 but incorporates seven additional *S. pneumoniae* serotypes [13]. Its ability to provide expanded serotype coverage with a single dose represents a significant advancement in pneumococcal disease prevention [13].

This paper aims to provide structured, evidence-informed, and specialty-specific expert opinions for the use of PCV20 in Indian adults. It is a narrative, expert-opinion–based document, not a formal guideline, and is intended to complement national guidelines.

## 2. Materials and Methods

### 2.1. Expert Selection

Experts were identified and invited based on their recognized clinical expertise, research contributions, and experience in managing adult patients at risk of pneumococcal disease in India. A scientific steering committee comprising senior clinical experts was constituted to oversee the scientific development and conduct of the expert panel. The steering committee identified and selected potential experts based on their recognized clinical expertise, research contributions, and experience in managing adults at risk of pneumococcal disease in India. The selected experts were subsequently invited to participate in the expert panel. Panelists were eligible if they were in active clinical practice in a relevant specialty, had a track record of peer-reviewed research and/or contributions to guidelines in adult immunization or the care of adults at risk of pneumococcal disease, and had substantial clinical experience in this field. The final panel comprised the 25 practicing clinicians who are the non-industry co-authors of this paper; all 25 experts who were approached agreed to participate. Representation was ensured across key specialties, including pulmonology, nephrology, microbiology, infectious diseases, internal medicine, endocrinology, oncology/hematology, preventive and social medicine, rheumatology, and cardiology. Geographic representation was sought across the northern, southern, eastern, and western regions of India, with panelists drawn from centers including Srinagar, Delhi, Gurugram, Chandigarh, Lucknow, Karnal, Kolkata, Mumbai, Pune, Chennai, Vellore, Hyderabad, Bengaluru, Cochin, and Thrissur. The sponsor’s Medical Affairs team supported the organization and convening of the meeting but had no role in the identification, selection, or invitation of the expert panel. The sponsor’s Medical Affairs team also, as named authors, contributed to the conceptualization and drafting of the manuscript. However, the clinical recommendations and expert opinions were determined independently by the multidisciplinary expert panel. Two expert group meetings were conducted in April 2025 to review evidence and provide guidance on PCV20 recommendations across different specialties.

### 2.2. Literature Review

To support evidence-informed discussions, a narrative (non-systematic) literature review was conducted. The search covered English-language publications from 2010 to 2025 in MEDLINE (PubMed), Google Scholar, and the Cochrane Library. Search terms included “pneumococcal vaccination”, “PCV13”, “PPSV23”, “PCV20”, “adult vaccination”, “immunobridging”, “efficacy”, “effectiveness”, “immunogenicity”, “safety”, “guidelines”, “chronic obstructive pulmonary disease”, “asthma”, “interstitial lung diseases”, “post-tuberculosis sequelae”, “coronavirus disease 2019”, “diabetes mellitus”, “chronic heart disease”, “autoimmune inflammatory rheumatic diseases”, “rheumatoid arthritis”, “systemic lupus erythematosus”, “disease-modifying antirheumatic drugs”, “hematologic malignancies”, “solid organ malignancies”, “solid organ transplants”, “thrombocytopenia”, “chronic kidney disease”, “dialysis”, “risk groups”, “antimicrobial resistance”, “serotype”, “serotype distribution”, “serotype prevalence”, “serotype coverage”, “invasive pneumococcal disease”, “community-acquired pneumonia”, and “India”. Titles and abstracts were screened by members of the scientific steering committee, and potentially relevant full-text articles were then assessed for eligibility. Studies were selected on the basis of their contextual relevance to adult pneumococcal vaccination and clinical practice in India, with priority given to randomized controlled trials, systematic reviews and meta-analyses, real-world effectiveness and surveillance studies, national and international guidelines, and India-specific epidemiological and serotype-distribution data. Non–English-language publications, conference abstracts lacking sufficient methodological detail, and studies not relevant to the adult populations of interest were excluded. India-specific data on invasive pneumococcal disease, pneumococcal community-acquired pneumonia, and serotype distribution were specifically sought to inform the assessment of the potential clinical benefit of PCV20 in the target population. A preliminary search of this literature also informed the development of the pre-meeting survey. Further, as this was a narrative rather than a systematic review, a formal quality appraisal was not performed; instead, the type and directness of the supporting evidence for major expert opinions are summarized in Supplementary Table S1.

### 2.3. Pre-Meeting Survey

A structured web-based survey was administered before the meetings to obtain baseline insights and experiences regarding adult pneumococcal vaccination. The survey questionnaire was developed by the scientific steering committee, informed by a brief preliminary review of relevant published literature and existing guideline recommendations, and was reviewed internally for content coverage and clarity (content and face validity) prior to administration; a formal psychometric validation was not performed. The survey included both multiple-choice and open-ended questions covering the perceived need for higher-valency vaccines (e.g., PCV20), implications for current vaccine use, anticipated benefits of PCV20, vaccination considerations for previously vaccinated and unvaccinated adults, the need for updated Indian guidelines, and information gaps expected at the time of PCV20 introduction. Responses were anonymized and summarized. The pre-meeting survey indicated generally favorable views toward PCV20, with 84% of respondents considering that PCV20 offered better protection in risk groups and 88% indicating that they would recommend PCV20 to patients with no prior pneumococcal vaccination. Responses also identified areas of variation that warranted further discussion, including future reliance on PCV20 versus case-by-case decision-making (52% vs. 40%), use of PCV20 in previously vaccinated patients (64% would recommend PCV20 to all eligible patients), and the need for updated Indian guidelines before routine clinical use (51% vs. 37% who considered international guidelines sufficient). Clinical efficacy and clinical-trial data were the highest-ranked information priority (48%). These summaries were presented to participants at the beginning of the meetings to support structured discussions. The survey was intended to capture baseline perspectives and frame the subsequent discussion. The survey findings were used to provide context and identify areas for further exploration during the expert discussions. The survey was not designed as a formal voting or consensus exercise and therefore did not use predefined agreement thresholds.

### 2.4. Nominal Group Technique Process

Both meetings followed a structured Nominal Group Technique (NGT) framework:

#### 2.4.1. Idea Generation

Participants reviewed the survey findings and literature summary, including areas identified by the survey as having broad support and those showing variation or uncertainty. These areas were considered as discussion points during the meeting. Participants independently documented their recommendations on adult pneumococcal vaccination, the role of PCV20, risk-group prioritization, and clinical practice considerations in India.

#### 2.4.2. Round Robin Sharing

The experts presented their ideas sequentially in a Round Robin format. All suggestions were recorded verbatim without debate to ensure equal contribution and prevent dominance by any individual participant. Additionally, specialty-specific breakout sessions were used to facilitate focused discussion within the clinical discipline.

#### 2.4.3. Clarification

Specialty groups discussed the role of PCV20 from their respective perspectives, highlighting key considerations and practical challenges, and refining wording to avoid overlap. The discussion aimed to build an understanding rather than reach an agreement.

#### 2.4.4. Development of Expert Opinion Statements

After consolidation, the refined inputs were synthesized into draft expert opinion statements, which were then reviewed by the experts for accuracy, completeness and relevance to clinical practice.

#### 2.4.5. Approval of Expert Opinions

The final expert opinion statements were circulated to all experts for independent review to obtain their approval. The final expert opinion statements were approved by all participating experts.

#### 2.4.6. Synthesis of Insights

Findings from the NGT discussions, along with baseline perspectives identified through the pre-meeting survey and supporting literature, were synthesized into thematic recommendations. The survey findings served to frame areas of discussion, while the expert deliberations and literature evidence informed the development of the final expert opinion statements. Meeting proceedings were documented, analyzed, and integrated with supporting literature to develop this expert opinion paper.

#### 2.4.7. Data Handling

The experts were informed that the discussions were being recorded solely for transcription, analysis of the discussions, and documentation of expert deliberations. All audio recordings were preserved securely.

## 3. Discussion

### 3.1. Recent Advancements in the Pneumococcal Conjugate Vaccine (PCV) Landscape

A key advancement in the PCV landscape is the introduction of PCV20. Approved in the United States (US) in 2021 and in Europe in 2022, PCV20 is built on the foundation of PCV13, sharing a similar structure, formulation, and manufacturing process [12]. It includes all serotypes present in PCV13 and expands coverage by incorporating seven additional pneumococcal serotypes [13,14].

While PCV13 and PPSV23 continue to be available in India, PCV20, approved by the Central Drugs Standard Control Organization of India in March 2025, represents an important step forward because of its broad serotype coverage within a single dose administration [15] and potential to enhance disease prevention and public health impact [16]. A detailed summary of PCV20, PCV13, and PPSV23 is provided in Table 1 [16,17,18,19].

### 3.2. Relevance of the Seven Additional PCV20 Serotypes to the Pneumococcal Disease Burden in India

PCV20 incorporates all serotypes included in PCV13, along with seven additional serotypes (8, 10A, 11A, 12F, 15B, 22F, and 33F) that have been identified as significant contributors to pneumococcal disease [24] and associated with serious clinical outcomes, including antimicrobial resistance (AMR), meningitis, sepsis, and mortality [25]. The clinical relevance of the serotypes included in PCV20 is presented in Table 2 [19,26,27,28,29,30,31,32,33,34,35,36,37,38,39,40,41].

A study reported that among 12,055 US adults (aged ≥18 years) hospitalized with CAP between 2013 and 2016, PCV20-unique serotypes accounted for approximately 27% of cases, highlighting the proportion of disease not addressed by PCV13 [42]. In another analysis from 33 high-income countries, including older adults (primarily ≥65 years), 52,905 IPD cases reported that PCV20 covered an additional 32.9% of IPD cases beyond PCV13, including key serotypes 8, 22F, 12F, and 11A [43]. These findings highlight that vaccinating adults with higher-valency PCVs, such as PCV20, could significantly reduce serious IPD outcomes that persist despite current vaccination strategies [43]. In India, a study by Varghese et al. reported an additional 10% serotype coverage with PCV20 compared with PCV13, with PCV20 covering 74.7% of invasive isolates in adults aged over 50 years, compared with 64% for PCV13 [44]. Recent surveillance data from India show the emergence of non-vaccine serotypes such as 15B and 11A—serotypes included in PCV20—thereby conferring a potential advantage over PCV13 [44]. The serotypes 8, 10A, 11A, 12F, 15B/C, 22F, and 33F have been associated with antimicrobial non-susceptibility to penicillin and macrolides in clinical isolates, contributing to AMR [26]. By covering several of these additional serotypes, PCV20 could, in principle, contribute to reducing the burden of antimicrobial-resistant pneumococcal disease [45,46]; however, the available Indian datasets are small and do not provide serotype-level data, so this potential benefit remains to be confirmed by more comprehensive surveillance linking antimicrobial resistance patterns with the pneumococcal serotypes circulating in India.

Among the higher-valency conjugate vaccines, the 15-valent pneumococcal conjugate vaccine (PCV15) adds only serotypes 22F and 33F to the PCV13 formulation, whereas PCV20 incorporates seven additional serotypes (8, 10A, 11A, 12F, 15B, 22F, and 33F) [47]. By comparison, PCV20 provides broader serotype coverage in a single conjugate dose against the serotypes currently circulating in Indian adults, supporting its role in adult pneumococcal vaccination strategies.

### 3.3. Global Clinical Evidence Base for the Immunogenicity and Safety of PCV20

Phase 3 clinical trials conducted across diverse adult populations and geographic regions have evaluated PCV20 in adults aged ≥18 years, including pneumococcal vaccine-naïve individuals, adults previously vaccinated with PCV13 and/or PPSV23, and populations with stable chronic medical conditions or other risk factors for pneumococcal disease. Across these groups, PCV20 has consistently demonstrated robust immunogenicity against all 20 serotypes and a favorable safety and tolerability profile comparable to PCV13.

**Figure 1:**
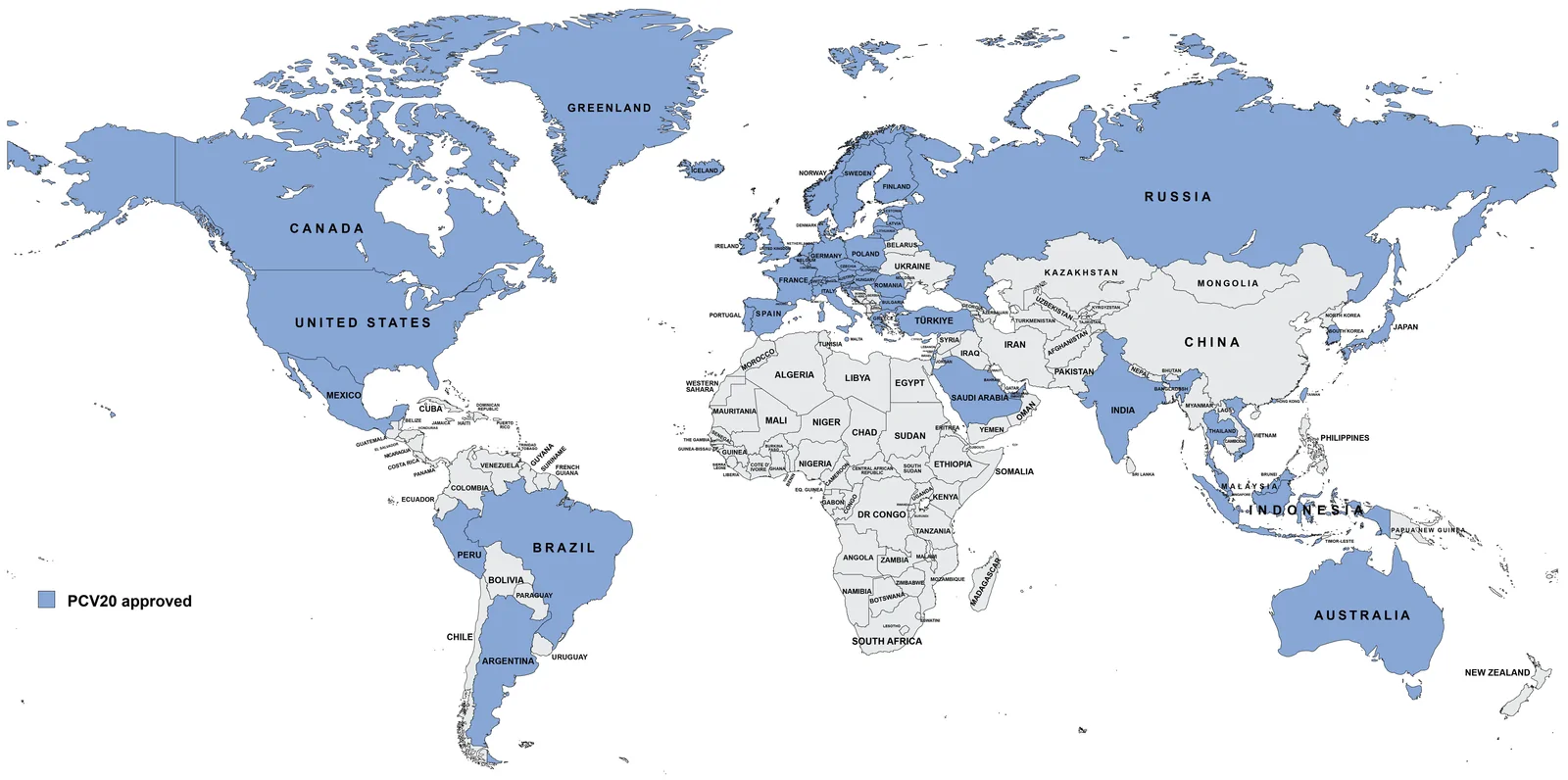
World map representing the global approval status of PCV20 for adult indication (based on the information available in public domain as of August 2025). Disclaimer: The world map is provided for illustrative purposes only and may not reflect current or officially recognized boundaries. The depiction of countries and borders does not imply any political or territorial position. Readers should refer to official sources for accurate information.

PCV20 has witnessed rapid global regulatory approvals following its initial approval in the US in June 2021 for the active immunization of adults against pneumonia and invasive disease caused by *S. pneumoniae* serotypes contained in the vaccine [20]. It was subsequently approved in the European Union in February 2022 [46] and is now approved in several countries [16,48,49,50,51,52,53,54,55,56,57,58,59,60,61,62,63,64,65,66,67,68,69,70,71,72,73], as presented in Figure 1.

Table 3 summarizes the key global clinical studies underpinning the evidence base for PCV20 across multiple regions and adult populations. PCV20 demonstrates consistent immunogenicity and safety across diverse adult populations, including vaccine-naïve individuals and those who have received previous vaccinations.

### 3.4. Safety and Immunogenicity of PCV20 in the Indian Adult Population

A recent phase 3, single-arm study evaluated the safety and immunogenicity of PCV20 in Indian pneumococcal vaccine-naïve adults aged ≥18 years (*n* = 400; 200 adults aged 18–49 years and 200 aged ≥50 years) [79]. The vaccine was well tolerated across both age groups, with no serious adverse events or deaths reported within one month after vaccination. Most local reactions and systemic events were mild or moderate in severity [79]. The most commonly reported solicited adverse reactions with PCV20 included pain and swelling at the injection site, muscle pain, fatigue, headache, and arthralgia [12], similar to those with PCV13, with no new safety concerns [22].

PCV20 vaccination also induced robust immune responses against all 20 vaccine serotypes, with adults aged 18–49 years and ≥50 years demonstrating substantial increases in serotype-specific opsonophagocytic activity geometric mean titers at one month after vaccination compared with pre-vaccination levels [79].

The clinical expectations for PCV20 are supported by an immunobridging approach, where the immunogenicity of PCV20 is compared with that of PCV13. As recognized by the World Health Organization (WHO) and regulatory authorities, immunobridging allows inference of expected vaccine efficacy based on comparable or superior immune responses, providing confidence in the protective benefits of PCV20 without the need for new large-scale efficacy trials [80,81].

#### Expert Opinion

PCV20 is well tolerated and elicits a strong immune response in Indian adults aged ≥18 years, with consistent safety and immunogenicity across all age groups.

### 3.5. Clinical Effectiveness and Cost-Effectiveness of PCV20 in Real-World Settings

Emerging real-world evidence on PCV20 complements the immunogenicity findings from clinical trials and suggests protection against pneumococcal disease in routine practice. In a US retrospective time-segment study (2022–2024), PCV20 demonstrated real-world effectiveness against IPD and all-cause pneumonia (ACP) in adults aged ≥65 years. Among 16.5 million adults, vaccine effectiveness against total IPD was 25.6% overall and 35.4%, 24.0%, and 16.6% in those aged 65–74, 75–84, and ≥85 years, respectively. Effectiveness against ACP was 15.2% overall, with corresponding estimates of 20.2%, 15.9%, and 12.5% across the age groups 65–74, 75–84, and ≥85 years, respectively. PCV20 was estimated to prevent 12.0 cases of IPD and 758 cases of pneumonia per 100,000 person-years, underscoring its population-level impact in routine clinical use [82]. The study reported PCV20 vaccine effectiveness of 15.2% against ACP, which is higher than observational estimates reported for PCV13 of 4.7–10.0% across different pneumonia outcomes, indicating an absolute incremental protection of approximately 5.2–10.5% with PCV20, likely reflecting its broader serotype coverage [82,83,84,85].

Similarly, real-world studies of PCV13 in US adults aged ≥65 years hospitalized with CAP reported strong real-world protection against vaccine-type (VT) disease. Adjusted analyses demonstrated vaccine effectiveness of 72.8% for all VT-CAP and 70.1% for non-bacteremic VT-CAP, highlighting the substantial reduction in the risk of PCV13-serotype CAP following its introduction into the national immunization program [86].

**Figure 2:**
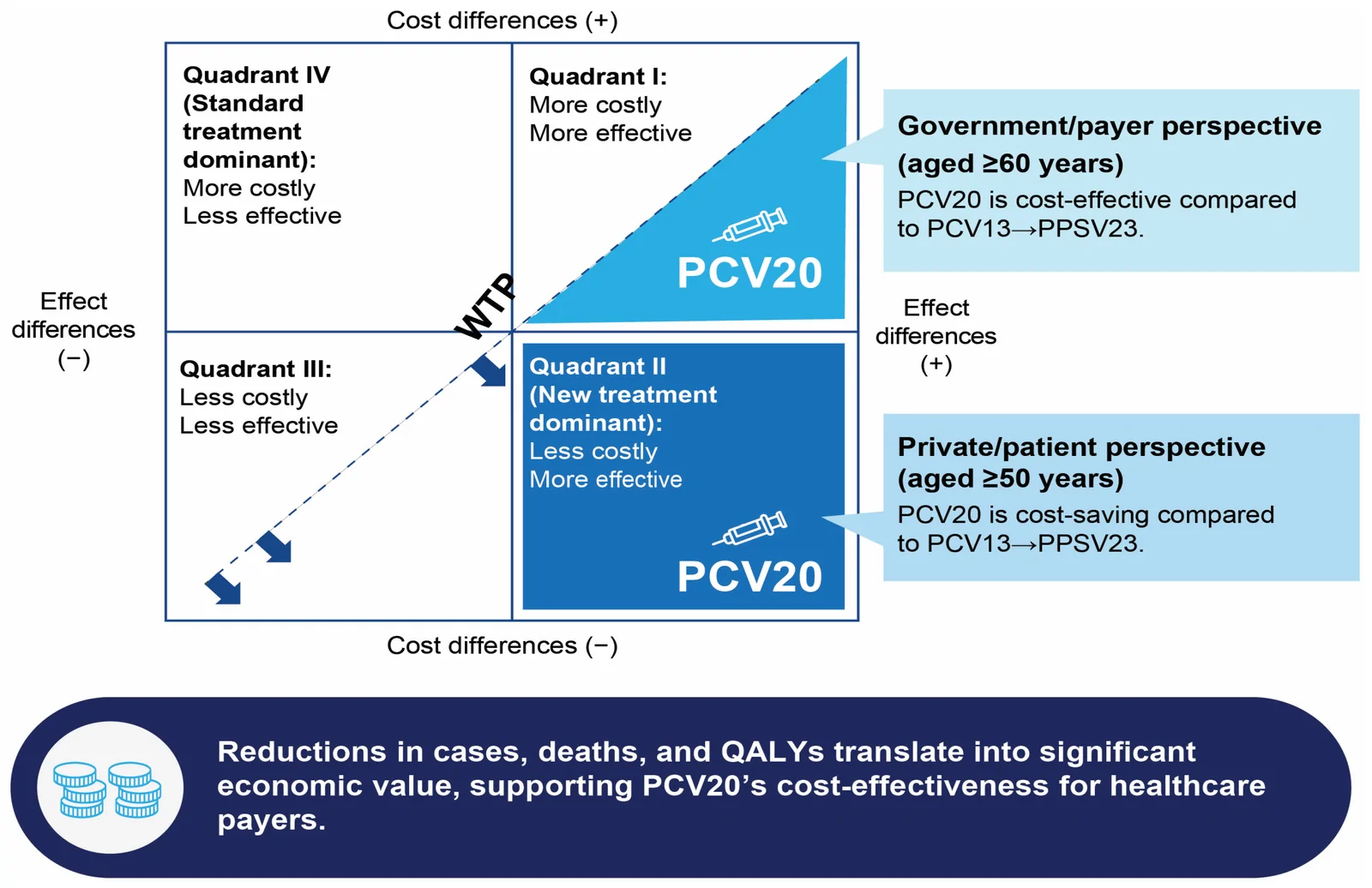
Cost-effectiveness of PCV20. PCV13: 13-valent pneumococcal conjugate vaccine; PCV20: 20-valent pneumococcal conjugate vaccine; PPSV23: 23-valent pneumococcal polysaccharide vaccine; QALY: Quality-Adjusted Life Year; WTP: Willingness to pay.

Economic modelling analyses in adult populations suggest that PCV20 vaccination may be associated with reductions in overall healthcare costs compared with existing pneumococcal vaccination strategies; however, these estimates are model-based, rely on setting-specific assumptions, and may not be directly transferable across healthcare systems. In a United Kingdom (UK) cost-effectiveness model that included adults aged ≥65 years and those aged 18–64 years with comorbidities, replacing PPSV23 with PCV20 was estimated to additionally avert 7789 cases of IPD and 140,046 cases of hospitalized CAP, prevent 22,199 deaths, and generate incremental savings of approximately £160 million, indicating that using PCV20 is an economically dominant strategy. Compared with current PPSV23-based recommendations in England, PCV20 was projected to prevent more hospitalizations, save more lives, and incur lower overall costs [87]. More recently, an updated societal-perspective analysis in England further supported these findings, demonstrating that PCV20 use among adults aged ≥65 years and adults aged 18–64 years with underlying risk conditions is cost-saving and economically dominant compared with PPSV23-based strategies, yielding a substantial net benefit to society [88]. In an economic evaluation study conducted in Argentina among moderate-/high-risk adults aged 18–64 years and all adults aged ≥65 years, replacement of PCV13 → PPSV23 with PCV20 was projected to additionally prevent 3838 cases of IPD, 10,380 cases of non-bacteremic pneumonia, and 1865 pneumococcal-related deaths, while resulting in net healthcare cost savings of $87.6 million [58]. In an economic evaluation study among at-risk and high-risk adults in India, PCV20 was modelled to be cost-saving compared with the sequential PCV13 → PPV23 strategy from the private payer perspective (incremental cost −₹2400 million; +11,385 quality-adjusted life years [QALYs]) and remained cost-effective compared with PPV23 alone (₹189,982 per QALY gained) and PCV13 alone (₹659,591 per QALY gained) in adults aged ≥50 years. From the government payer perspective in adults aged ≥60 years, PCV20 was also cost-effective compared with PCV13 → PPSV23 (₹577,810 per QALY gained). A quadrant-based incremental cost-effectiveness ratio plane representing the cost-effectiveness of PCV20 in India is depicted in Figure 2 [89] (preliminary data from Taur, S. et al. have been presented as a poster at the ISPOR Europe 2025 conference; a full peer-reviewed publication of these findings is anticipated). As these estimates are derived largely from economic models including a preliminary India-specific analysis, they should be interpreted with caution.

#### Expert Opinion

PCV20 is an effective adult pneumococcal vaccine which has demonstrated real-world protection against IPD and pneumonia and is projected, in modelling studies, to be cost-saving or cost-effective compared with existing strategies (PCV13 → PPSV23).

### 3.6. Patient Populations Prioritized for PCV20 Vaccination

**Figure 3:**
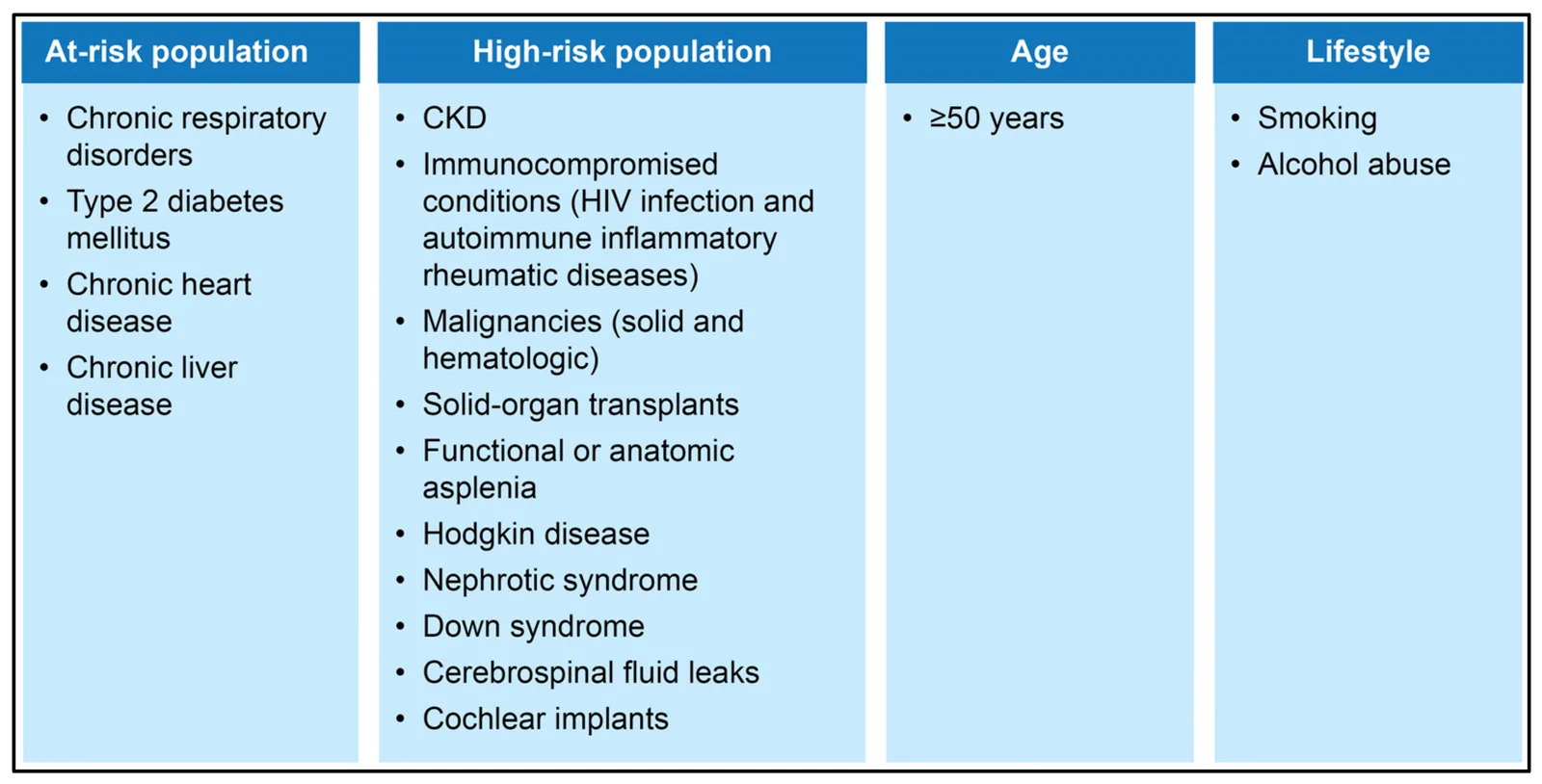
Patients prioritized for PCV20 vaccination. CKD: Chronic kidney disease; HIV: Human immunodeficiency virus; PCV20: 20-valent pneumococcal conjugate vaccine.

Priority populations for PCV20 vaccination include all adults aged ≥50 years, irrespective of underlying risk factors, as well as adults aged 18–49 years with at-risk or high-risk medical conditions [90]. At-risk and high-risk patient populations, along with risk factors such as age and lifestyle, are summarized in Figure 3 [91]. Within this framework, the panel distinguished established, guideline-based indications for adult pneumococcal vaccination from additional risk groups it proposed for consideration in the Indian context; the latter are not recognized as stand-alone indications in major international guidelines and should be vaccinated on the basis of age and underlying medical risk rather than the exposure itself (Table 4).

### 3.7. Guidance on the Use of PCV20 in Indian Adults, Including Vaccine-Naïve and Previously Vaccinated Individuals

Current recommendations for PCV20 use, including guidance from the national and international guidelines, are summarized in Supplementary Table S2 [9,90,92,93,94,95,96,97,98,99]. Updated national and international guidelines have lowered the recommended age for pneumococcal vaccination from ≥65 years to ≥50 years, underscoring the need for proactive vaccination strategies in adults [100]. The potential role of PCV20 in the management of adults at risk of pneumococcal disease in the Indian setting was discussed, with input from experts across multiple specialties. A detailed overview of the recommendations for PCV20 in adults, based on expert opinion and available evidence, is provided in Table 4 and Supplementary Figure S1.

### 3.8. Rationale for the Single-Dose Recommendation of PCV20

PCV20 is a conjugate vaccine that induces a T-cell-dependent immune response, leading to the formation of long-lived plasma cells and immunological memory, thereby providing sustained protection following a single dose. Unlike polysaccharide vaccines, conjugate vaccines elicit robust memory B-cell responses, supporting long-term immunity without the need for routine booster doses in adults [17].

In addition to these immunologic advantages, PCV20, currently available in India, offers broad serotype coverage, including all serotypes contained in PCV13 plus seven additional serotypes associated with invasive disease in adults, providing coverage comparable to the sequential PCV13 → PPSV23 regimen [91]. This combination of sustained immune memory and expanded serotype coverage supports the adequacy of a single-dose PCV20 strategy for adult pneumococcal protection.

In the CAPiTA trial, a single dose of PCV13 demonstrated sustained efficacy against VT pneumococcal CAP and IPD over a mean follow-up of approximately four years, with no evidence of waning protection during the observation period [101]. These findings reinforce the concept that T-cell-dependent immune responses induced by PCVs can provide long-lasting clinical protection following a single adult dose, supporting extrapolation of durable protection to higher-valent conjugate vaccines such as PCV20.

**Figure 4:**
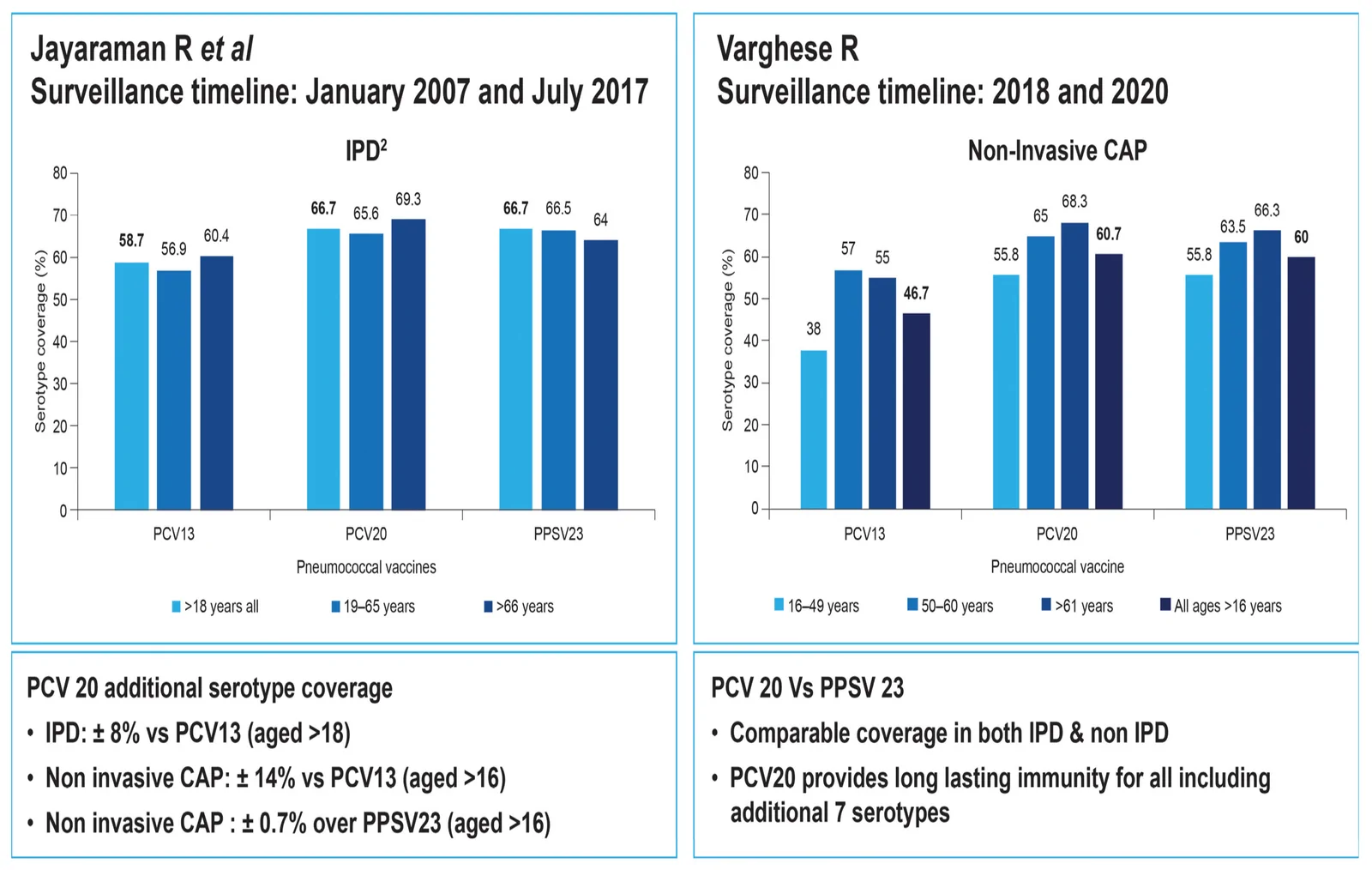
Serotype coverage by pneumococcal vaccines in India [19,102,103,104]. CAP: Community-acquired pneumonia; IPD: Invasive pneumococcal disease; PCV13: 13-valent pneumococcal conjugate vaccine; PCV20: 20-valent pneumococcal conjugate vaccine; PPSV23: 23-valent pneumococcal polysaccharide vaccine.

PCV20 offers comparable serotype coverage to PPSV23 and improved coverage relative to PCV13, as supported by data from India (Figure 4) [19,102,103,104].

Taken together, the T-cell-dependent immune response induced by PCV20, its ability to generate immunological memory with sustained protection, and its broad serotype coverage comparable to PPSV23 support the use of a single-dose PCV20 strategy as an appropriate approach for adult protection against pneumococcal disease.

### 3.9. Clinical Considerations for Using PCV20 in Patients with Thrombocytopenia or Coagulation Disorders

Intramuscular administration may cause bleeding and, therefore, requires caution in individuals with thrombocytopenia or bleeding disorders. In patients with coagulation disorders, clinicians should carefully evaluate bleeding risk before proceeding with intramuscular injections and consider subcutaneous administration when the clinical benefit outweighs potential risks [46]. In line with this, the UK Patient Group Direction (PGD) emphasizes that individuals with stable bleeding disorders or those on well-controlled anticoagulation (e.g., warfarin with an international normalized ratio within therapeutic range) can receive PCV20 intramuscularly if a clinician familiar with their bleeding risk deems it safe. Vaccination should ideally be scheduled shortly after hemostatic treatment, when applicable [105].

### 3.10. Key Clinical Factors for Pulmonologists Incorporating PCV20 into Practice

Patients with chronic pulmonary disease are at increased risk of pneumococcal pneumonia and IPD, making pneumococcal vaccination a key preventive consideration in pulmonology practice. The Indian consensus guideline on adult immunization recommends pneumococcal vaccination in adults with lung diseases [9]. According to the Global Initiative for Chronic Obstructive Lung Disease (GOLD) 2025 guidelines, all patients with chronic obstructive pulmonary disease (COPD) should receive a single dose of PCV20 [106]. As PCV20 is now approved in India, it can be administered to patients with COPD in the Indian population in accordance with the GOLD 2025 guidelines.

Many chronic pulmonary conditions, particularly interstitial lung diseases (ILDs), including idiopathic pulmonary fibrosis, idiopathic interstitial pneumonias, connective tissue disease-related interstitial pneumonia, drug-induced ILD, and chronic hypersensitive pneumonia, are commonly managed with systemic corticosteroids or immunomodulating therapies (such as methotrexate or anti-tumor necrosis factor agents). Evidence indicates that PCV13 and PPSV23 may be administered to patients with ILD receiving corticosteroids or other immunosuppressive therapy, as evidence shows preserved vaccine responsiveness in this population. However, when clinically appropriate, tapering high-dose corticosteroids (Table 5) should be considered, given their potential to attenuate pneumococcal antibody responses and the likelihood of improving immunogenicity with dose reduction [107,108].

Additionally, the Global Initiative for Asthma (GINA) 2025 guidelines advise people with asthma to stay up to date on respiratory vaccines, including pneumococcal vaccines, as recommended by local guidelines for their age group [110]. In this context, PCV20 may be considered, given its recent approval in India as a higher-valent conjugate vaccine for adults ≥18 years and its recommendation in Indian guidelines [9].

Pneumococcal vaccination is a critical preventive strategy for individuals with post-tuberculosis lung disease (PTLD), as residual structural lung damage places them at ongoing risk for bacterial pneumonia. Accordingly, current recommendations support administering a single dose of PCV20 in adults aged ≥18 years with PTLD [111].

Clinical evidence with PCV13 demonstrates clinical benefits in adult populations at risk for pneumococcal disease. In the HOPE COPD study, among patients hospitalized with acute COPD exacerbations, those previously vaccinated with PCV13 had improved clinical outcomes compared with unvaccinated patients, including a lower proportion requiring assisted ventilation (43.3% vs. 60%; *p* = 0.04), shorter ICU stays (0.67 days vs. 1.77 days), and reduced overall hospital stay (4.50 days vs. 5.47 days; *p* = 0.005) [112]. Similarly, the IMPROVE study, a 1-year observational study in pneumococcal vaccine–naïve COPD patients with prior hospitalization for exacerbations, showed that PCV13 vaccination significantly reduced the mean annual exacerbation rate (1.17 vs. 3.69; *p* < 0.0001), all-cause hospitalizations (0.71 vs. 1.60; *p* < 0.0001), and the occurrence of CAP [113]. Based on these findings, PCV20 is expected to provide comparable clinical benefits in adults, while offering broad serotype coverage.

#### Expert Opinion

PCV20 is advised for individuals with COPD, asthma, ILD, and PTLD, due to their heightened risk of pneumococcal disease.

### 3.11. Specialty-Specific Considerations for Using PCV20 in Patients with Chronic Kidney Disease (CKD), Including Those on Dialysis

The Kidney Disease Improving Global Outcomes 2021 guidelines recommend pneumococcal vaccination for patients with glomerular disease, nephrotic syndrome, and CKD [114]. Similarly, the US Centers for Disease Control and Prevention (CDC) Advisory Committee on Immunization Practices (ACIP) guidelines recommend pneumococcal vaccination for patients on hemodialysis [115,116]. The burden of pneumococcal disease in patients with CKD is substantial, whereas the cost of pneumococcal vaccination is low relative to overall healthcare expenditure in this population, with no evidence of clinically relevant disadvantages. Therefore, pneumococcal vaccination should be recommended for all patients with CKD, preferably early in the disease course [117]. Furthermore, given suboptimal seroconversion in patients on dialysis, pneumococcal vaccination of previously unvaccinated individuals with advanced CKD is recommended before the initiation of dialysis [118]. Expert opinions further emphasize vaccinating patients as early as possible in the disease course to prevent the risk of infections that may lead to disease progression and hospitalization.

#### Expert Opinion

PCV20 should be considered early in patients with CKD at all stages, including dialysis and nephrotic syndrome, to reduce pneumococcal disease risk.

### 3.12. Considerations for Using PCV20 in Adults with Diabetes Mellitus

The timing of vaccination and glycemic status are important clinical considerations when administering PCV20.

Evidence suggests that the optimal window for pneumococcal immunization in adults with diabetes is within the first six months after diagnosis, given the burden of comorbidities and risk factors. Vaccination may also be considered beyond six months to allow time for medication adjustments and stabilization of glycemic status [119]. Although improved glycemic control reduces diabetic complications and supports immune function, pneumococcal vaccination should be administered regardless of glycemic control [119]. Clinical outcomes highlight the need to prioritize pneumococcal vaccination in patients with diabetes mellitus. A post hoc analysis of the CAPiTA study (84,496 immunocompetent adults aged ≥65 years) showed significantly higher protection against VT-CAP among participants with diabetes (89.5%) vs. those without (24.7%), confirming diabetes as a significant effect modifier (*p* = 0.002) of vaccine effectiveness [120]. This highlights the relevance of the pneumococcal vaccine for patients managed in endocrinology practice.

#### Expert Opinion

PCV20 can be administered to all adults with diabetes mellitus (type 1 diabetes mellitus and type 2 diabetes mellitus), irrespective of their glycemic status and duration of diabetes.

### 3.13. Importance of Pneumococcal Protection with PCV20 for Adults with Cardiac Disease

A prospective observational cohort study by Corrales-Medina et al. reported that incident cardiac complications, such as new or worsening heart failure, arrhythmias, and myocardial infarction, were common among patients with CAP (26.7% in inpatients) and were associated with increased short-term mortality. These incident cardiac complications were associated with an increased risk of 30-day mortality (odds ratio: 1.6; 95% confidence interval: 1.04–2.5) [121]. Given this risk, pneumococcal vaccination constitutes an important preventive strategy for adults with underlying cardiac disease to reduce the risk of pneumonia and its associated cardiovascular complications.

Evidence from direct clinical trials on PCV20’s role in reducing cardiovascular events is currently unavailable [122,123]. A meta-analysis showed that pneumococcal vaccination led to a significant reduction in all-cause mortality (hazard ratio: 0.78, *p* < 0.001) among adults with cardiovascular disease or those at very high cardiovascular risk. Overall, pneumococcal vaccination was associated with a 22% decrease in all-cause mortality in this population [123].

The 2025 European Society of Cardiology consensus statement considers vaccination, including pneumococcal vaccination, a key component of cardiovascular disease prevention [124]. According to the 2025 Concise Clinical Guidance by the American College of Cardiology, a single dose of PCV20 is recommended for adults aged 19 years or older with heart disease to protect against pneumonia, bacteremia, meningitis, and associated risks of hospitalization and death [125]. The 2025 Heart Failure Association of India Guidelines for Diagnosis and Management of Heart Failure recommend administering a conjugated pneumococcal vaccine to all patients with heart failure without known contraindications [126].

Together, these findings highlight the importance of PCV20 vaccination in adults with cardiac conditions to help prevent pneumococcal infections and their potential cardiovascular complications.

#### Expert Opinion

PCV20 immunization is advised for adults with chronic cardiac conditions (chronic heart disease, including congestive heart failure and cardiomyopathies) to protect against pneumococcal disease.

### 3.14. Timing of PCV20 Vaccination for Adults with Rheumatic and Musculoskeletal Diseases (RMDs) Receiving Immunosuppressive Therapy

As per the 2022 American College of Rheumatology guideline for vaccinations of patients with RMDs, pneumococcal vaccination is strongly recommended for patients with RMDs aged <65 years who are receiving immunosuppressive medication. However, in such patients, the timing of vaccination is crucial to achieve an optimal immune response. As PCV20 is a non-live vaccine, it can generally be administered during immunosuppressive therapy, with specific considerations depending on the medication used. Table 6 presents recommendations for medication management at the time of non-live vaccine administration in this population. Much of this guidance is extrapolated from recommendations for non-live vaccines in general rather than PCV20-specific data; because different immunosuppressive agents have markedly different effects on vaccine immunogenicity, the timing of vaccination should be individualized to the specific therapy. For patients on corticosteroids, Table 5 provides the details of PCV20 vaccination as per the dose.

#### Expert Opinion

Where possible, PCV20 should be administered before initiating immunosuppressive therapy. In patients about to start rituximab, PCV20 should be given at least 2–4 weeks before the first dose; in those already receiving rituximab, vaccination should be timed six months after the last rituximab dose—with rituximab withheld for at least 2 weeks after vaccination where clinically feasible.

PCV20 can be administered during methotrexate therapy or other ongoing immunosuppressive treatments, including corticosteroids. However, PCV20 should be deferred in individuals receiving high-dose corticosteroids (≥20 mg/day of prednisolone or equivalent dose) until the dose is reduced below this level.

### 3.15. Key Considerations for Administering PCV20 in Patients with Hematological and Solid Organ Malignancies

According to the National Comprehensive Cancer Network guidelines for hematological and solid organ malignancies, vaccination can significantly lower the risk of infection-related illness and death in patients with cancer. For newly diagnosed adults with hematological and solid organ malignancies, PCV may be provided after assessing the immune status. PCV20 is recommended for patients with cancer who have not received prior pneumococcal vaccination. There is no need for an additional PPSV23 dose if PCV20 is administered. Individuals previously vaccinated with PPSV23 are eligible to receive PCV20. Patients who have received only PCV13 can receive PCV20 instead of PPSV23, provided they have received PCV13 at least one year before. If the patient has received PCV13 and 1–2 doses of PPSV23, PCV20 may be administered five years after the most recent dose of PPSV23 [95].

Consistent with this approach, the 2024 American Society of Clinical Oncology guidelines recommend that, when feasible, any vaccination should ideally be administered 2–4 weeks before the initiation of planned cancer therapy. Nevertheless, non-live vaccines such as the pneumococcal vaccine can be safely administered during or after chemotherapy [96].

According to the UK PGD, for patients scheduled to receive chemotherapy, PCV20 should ideally be administered 2–4 weeks before treatment initiation to optimize the immune response. If pre-treatment vaccination is not feasible, vaccination may be given during chemotherapy, recognizing the potential for reduced immunogenicity, and revaccination may be considered after completion of therapy and immune recovery. If vaccination has not been given before chemotherapy, immunization is preferably deferred until at least three months after completion of chemotherapy; however, vaccination should not be delayed if there is a risk that the patient may not receive the vaccine at a later time [105].

The pneumococcal vaccine is ideally administered before starting solid organ cancer therapy, with the time of diagnosis being the preferred period to ensure optimal antibody development. Vaccination during active cancer treatment is not recommended. For patients who remain unvaccinated, the vaccine should be given at least three months after completing therapy, as immune function can remain compromised for up to six months. In high-risk individuals, the CDC advises an additional dose of pneumococcal vaccine, with a minimum interval of five years between doses [127].

#### Expert Opinion

In adults with hematological malignancies, PCV20 should ideally be given 2–4 weeks before initiating chemotherapy; however, it can also be administered during chemotherapy or at least three months after the completion of chemotherapy.

In adults with solid organ malignancies, it is recommended to administer PCV20 at the time of cancer diagnosis, before initiating treatment (chemotherapy or radiation therapy), to ensure an optimal immune response.

### 3.16. Key Considerations for Administering PCV20 in HSCT and Solid Organ Transplant Recipients

The PCV20 vaccine should be administered before HSCT, at least two weeks before the conditioning regimen, because pre-transplant vaccination may provide protection during the peri-transplant period, including conditioning and the immediate post-transplant phase, despite the anticipated loss of vaccine-induced immunity after HSCT [128].

Further, pneumococcal vaccination should be initiated with a PCV20 three months after the transplant [128]. Its immunization series is recommended as a 3+1 schedule. The primary series consists of three doses, starting 3–6 months after HSCT, with intervals of at least four weeks. A booster (fourth) dose is recommended six months after completing the primary series [46,48,127]. However, vaccine administration should be avoided in case of severe illness or fever and should be given once the patient is stable [128].

In solid organ transplants, such as lung, heart, kidney, or liver transplants, PCV20 should generally be administered at least two weeks before the procedure.

#### Expert Opinion

PCV20 should be administered to HSCT recipients at least two weeks before transplantation, when feasible, or starting three months after HSCT as a three-dose series given at four-week intervals, followed by a booster at six months after the primary vaccination series.

In solid organ transplant recipients, PCV20 should preferably be administered at least two weeks before transplantation, or, if deferred, 3–6 months after transplantation, as a single dose.

### 3.17. Recommended Interval Between Pneumococcal Conjugate Vaccines, Especially in Patients with High-Risk Conditions

Traditionally, high-risk adults and at-risk adults who previously received PCV13 were advised to receive PPSV23 eight weeks later and one year later, respectively [9]. With the availability of PCV20, the US CDC ACIP guidance recommends administering PCV20 ≥1 year after PCV13, effectively replacing PPSV23 in this sequence [90].

In a pivotal phase 3 study by Cannon et al. (2021), adults aged ≥65 years with varied prior pneumococcal vaccination histories demonstrated robust immune responses to all 20 serotypes one month after PCV20, irrespective of previous vaccination status [75].

Supporting this concept, earlier studies by Essink et al. (2022) and Hurley et al. (2021) showed that shorter-than–recommended intervals between pneumococcal vaccines resulted in strong immunogenicity and comparable safety across vaccine groups, with no vaccine-related serious adverse events [17,69]. Although these studies primarily evaluated PCV13–PPSV23 sequences, these data were largely generated with PCV13–PPSV23 schedules and should not be directly extrapolated to the PCV13–PCV20 sequence.

The flexibility in sequencing is consistent with global adult pneumococcal vaccination policies, where most national recommendations include sequential use of PCVs and PPSVs, while allowing adaptation of timing based on local risk profiles and population demographics [129].

In line with current CDC ACIP guidelines 90], an interval of ≥1 year between PCV13 and PCV20 should be retained. In the absence of specific evidence supporting a shorter PCV13–PCV20 interval, deviation from this recommended interval is not advised; the timing of vaccination may nonetheless be individualized within this framework according to patient risk, the local burden of pneumococcal disease, and the urgency of protection.

#### Expert Opinion

In high-risk adults and at-risk adults who previously received PCV13, PCV20 should be administered ≥1 year after PCV13, consistent with current CDC ACIP guidance. This interval should be retained; however, the exact timing may be individualized according to patient risk factors and clinical judgment.

### 3.18. Essential Components of Patient Counseling to Enhance Understanding, Acceptance, and Adherence to PCV20 Vaccination

Effective counseling for PCV20 should combine clear communication of personal risk, a strong provider recommendation, and simple, practical guidance. Clinicians should explain the real consequences of remaining unvaccinated, such as recurrent pneumonia, hospitalizations, or invasive infections, as these concrete risks motivate patients more than abstract probabilities [130]. A recommendation from healthcare professionals (HCPs) remains the strongest predictor of adult vaccine uptake [130].

Counseling should also correct misconceptions and address concerns linked to pandemic-era vaccine hesitancy [131,132]. Highlighting PCV20’s single-dose schedule and broad serotype coverage can simplify decision-making and improve acceptance [91]. Economic analyses show that PCV20 alone is cost-effective or dominant in most adult vaccination scenarios [133]. Reminder recall systems, follow-ups, and integration of vaccination into routine chronic disease care can further increase uptake [134]. Opportunistic counseling during respiratory illnesses and strong HCP engagement, consistently shown to drive vaccination behavior, are also essential [135,136]. Overall, counseling should make vaccination clear, trusted, convenient, and personally relevant to each patient.

The CDC SHARE framework (Share, Highlight, Address, Remind, and Explain) guides effective vaccine counseling. Clinicians should share personalized reasons for vaccination, highlight positive experiences, address patient concerns, remind patients of the protection vaccines provide, and explain the potential health, time, and financial consequences of remaining unvaccinated [136].

### 3.19. Contraindications and Safety Measures to Assess Before Recommending PCV20 in Adults

PCV20 is contraindicated in individuals with a severe allergic reaction (e.g., anaphylaxis) to any component of the vaccine or to diphtheria toxoid [23].

Although thrombocytopenia is not an absolute contraindication, PCV20 should be used with caution in individuals with thrombocytopenia or bleeding disorders, as intramuscular administration may increase the risk of bleeding [46]. Vaccination should be deferred in individuals with acute severe febrile illness [46,48].

Adequate and well-controlled studies of PCV20 in pregnant women are not available, and existing data are insufficient to determine vaccine-associated risks during pregnancy. In preclinical animal studies, no adverse effects on preweaning development and no vaccine-related fetal malformations were observed [23]. Despite reassuring animal data, administration of PCV20 during pregnancy should be considered only when the potential benefits outweigh potential risks to the mother and fetus [46].

### 3.20. Recommended Storage Conditions and Shelf-Life Requirements for PCV20

PCV20 should be stored under refrigerated conditions at 2 °C to 8 °C. Syringes are recommended to be stored horizontally to reduce resuspension time before administration. The vaccine must not be frozen [23]. PCV20 has a shelf-life of 24 months [48].

### 3.21. Study Limitations

The pre-meeting survey provided limited methodological detail, which reduced the strength of its interpretations. As the exercise used NGT rather than a formal consensus method (Delphi), its rigor is inherently different. The absence of structured individual voting and predefined agreement thresholds for the expert opinions limited the quantitative assessment of agreement among experts. The evidence base is also limited owing to scarce Indian data on PCV20 and reliance on extrapolation from PCV13 and PPSV23 studies. Furthermore, the study did not formally assign levels of evidence or grades to the opinions. Additionally, the paper does not fully explore local implementation challenges such as rural access, cost considerations, and variations in provider awareness of pneumococcal vaccination guidelines. In terms of transparency, the expert panel was assembled purposively on the basis of clinical and research expertise rather than by random sampling of specialists, which may introduce selection bias and may limit the generalizability of the opinions. Similarly, although the supporting literature was identified through a structured search, this was a narrative rather than a systematic review, and the selection and weighting of evidence involved expert judgment; relevant studies may therefore have been missed, and the recommendations should be interpreted as evidence-informed expert opinion rather than as a formal evidence synthesis.

### 3.22. Summary of Specialty-Specific PCV20 Expert Opinions

The specialty-specific expert opinions arising from the multidisciplinary deliberations are consolidated in Table 7 to support their application in routine clinical practice.

## 4. Conclusions

Based on the available safety and immunogenicity data, the experts acknowledged PCV20 as a valuable option for adult immunization. Its broad serotype coverage and single-dose schedule may simplify adult vaccination and could improve adherence relative to sequential regimens. Early prevention is especially important in India, where comorbidities appear earlier in adulthood. PCV20 aligns with national and international recommendations, and the panel supported a single dose for adults aged ≥50 years and for at-risk or high-risk adults aged 18–49 years. These are multidisciplinary, evidence-informed expert opinions intended to complement, not replace, the formal national recommendations; they should be interpreted in light of the remaining uncertainty regarding real-world effectiveness, implementation, affordability, and the limited availability of India-specific PCV20 data.

Improving uptake will require tailored strategies, as affordability remains a key barrier. With supportive policies and patient education, PCV20 offers a practical opportunity to reduce pneumococcal disease burden in India.

## Figures and Tables

**Table 1:** Overview of PCV20, PCV13, and PPSV23 with respect to vaccine type, mechanism of action, administration and dosage, indications, and regulatory approval in adults.

Aspect	PCV20	PCV13	PPSV23
Vaccine type	Polysaccharide conjugate vaccine	Polysaccharide conjugate vaccine	Polysaccharide vaccine
Mechanism of action	Elicits a T-cell-dependent response with the establishment of B-cell memory and long-term immunization	Elicits a T-cell-dependent response with the establishment of B-cell memory and long-term immunization	Produces a T-cell-independent response, without the establishment of B-cell memory
Administration and dosage	Route: Intramuscular injection Dosage: 0.5 mL	Route: Intramuscular injection Dosage: 0.5 mL	Route: Intramuscular or subcutaneous injection Dosage: 0.5 mL
Indication	Active immunization for the prevention of invasive disease and pneumonia caused by *S. pneumoniae* serotypes 1, 3, 4, 5, 6A, 6B, 7F, 8, 9V, 10A, 11A, 12F, 14, 15B, 18C, 19A, 19F, 22F, 23F, and 33F in individuals 18 years of age and older	Active immunization for the prevention of pneumonia and invasive disease caused by *S. pneumoniae* serotypes 1, 3, 4, 5, 6A, 6B, 7F, 9V, 14, 18C, 19A, 19F, and 23F in adults 18 to 49 years of age and in adults 50 years and older age group	Active immunization for the prevention of pneumococcal disease caused by the 23 serotypes contained in the vaccine (1, 2, 3, 4, 5, 6B, 7F, 8, 9N, 9V, 10A, 11A, 12F, 14, 15B, 17F, 18C, 19F, 19A, 20, 22F, 23F, and 33F) in individuals 50 years of age or older and individuals aged ≥2 years who are at increased risk for pneumococcal disease
Indian regulatory authority (CDSCO) approval	2025	2011	1996
Non-invasive pneumonia [20,21]	Yes	Yes	Variable
Hypo-responsiveness to multiple doses [20,21]	No/NA	No/NA	Yes >2 doses
Nasopharyngeal carriage reduction [21]	Yes	Yes	No

CDSCO: Central Drugs Standard Control Organization; NA: Not applicable; PCV13: 13-valent pneumococcal conjugate vaccine; PCV20: 20-valent pneumococcal conjugate vaccine; PPSV23: 23-valent pneumococcal polysaccharide vaccine; *S. pneumoniae*: *Streptococcus pneumoniae* [16,17,22,23].

**Table 2:** Serotypes included in PCV20 and their clinical relevance.

	PCV 20 Serotypes [19]																			
	4	6B	9V	14	18C	19F	23F	1	5	7F	3	6A	19A	22F	33F	8	10A	11A	12F	15B
Mortality/CFR [26,27,28,29,30]			●	●		●	●	●	●		●	●	●	●	●	●	●	●	●	●
ICU admissions [30,31,32]				●			●	●			●		●			●			●	
Virulence ^†^ [30]				●	●	●	●	●	●	●	●	●	●	●	●	●	●	●	●	●
AMR [26,33,34,35,36]		●	●			●						●	●	●	●		●	●	●	●
Outbreaks [37,38,39,40,41]	●		●	●	●			●	●	●			●			●	●		●	

^†^ Virulence was assessed among the 24 serotypes detected by UAD (1, 3, 4, 5, 6A, 7F, 6B, 9V, 14, 18C, 19A, 19F, 23F, 22F, 33F, 8, 10A, 11A, 12F, 15B/C, 2, 9N, 17F, and 20). ● Indicates a reported association between the pneumococcal serotype and the corresponding characteristic, based on the cited literature. Absence of a (●) does not necessarily indicate absence of an association. AMR: Antimicrobial resistance; CFR: Case fatality rate; ICU: Intensive care unit; PCV20: 20-valent pneumococcal conjugate vaccine; PSI: Pneumonia severity index; UAD: Urinary antigen detection.

**Table 3:** Overview of global clinical evidence base for PCV20 in adults.

Author	Study	Patient Population	Patient Comorbidities	Pneumococcal Vaccine-Naïve Adults	Intervention	Comparison	Findings
Essink et al., 2022 [69]	Pivotal phase 3, randomized clinical trial	3902 adults; ≥18 years (18–49, 50–59, and ≥60 years)	Healthy participants or those with a stable underlying disease	Yes	PCV20	PCV13, PCV13 → PPSV23	PCV20 demonstrated immunogenicity comparable to PCV13 for shared serotypes and for six additional PPSV23 serotypes and was well-tolerated with a favorable safety profile.
Klein et al., 2021 [74]	Phase 3, randomized, double-blind study	1710 adults; 18–49 years of age	Individuals with preexisting stable disease **	Yes	PCV20	PCV13	PCV20 demonstrated robust and consistent immunogenicity comparable to PCV13. PCV20 was well tolerated. Rates of local reactions, systemic events, and adverse events were similar between PCV20 and PCV13 recipients.
Cannon et al., 2021 [75]	Phase 3, randomized, double-blind, multicenter study	875 adults; 65 years of age, with prior vaccination history (PPSV23, PCV13, or both PCV13 and PPSV23)	Adults with preexisting stable disease	No	PCV20	For safety comparisons In prior PPSV23 recipients: PCV20 vs. PCV13 In prior PCV13 recipients: PCV20 vs. PPSV23 In prior PCV13 → PPSV23 recipients: no comparator (single-arm PCV20) For immunogenicity comparisons No between-group comparisons performed; immune responses were assessed only in PCV20 recipients across all prior vaccination groups.	PCV20 elicited robust immune responses one month after administration across all prior vaccination groups. PCV20 was well tolerated. Rates of local reactions, systemic events, and adverse events were generally similar with PCV13 and PPSV23 across prior vaccination groups.
Sabharwal et al., 2022 [76]	Post hoc analysis of randomized, active-controlled, double-blind, phase 3 studies (two studies, Klein and Essink et al.)	1329 adults; ≥18 years of age	All participants had ≥1 risk factor *	Yes	PCV20	PCV13, followed by PPSV23 one month later	In at-risk adults, PCV20 was immunogenic and comparable to PCV13 for shared serotypes, while PPSV23 comparison was limited only to older adults (60–64 years), where PCV20 generally showed higher responses for most additional serotypes.
Cannon et al., 2023 [77]	Phase 3, randomized, double-blind, multicenter study	1796 adults; ≥65 years of age	Healthy participants or those with a stable preexisting disease	Mixed (vaccine-naïve and previously vaccinated)	PCV20 and QIV together, followed by a saline injection one month later	QIV and saline first, followed by PCV20 one month later	PCV20 and QIV coadministration showed a non-inferior response compared with separate administration. Simultaneous administration can be considered.
Hurley et al., 2021 [17]	Randomized, active-controlled, double-blinded trial	444 adults aged 60–64 years	Healthy participants, including those with a diagnosis of preexisting stable disease	Yes	PCV20, followed by a saline placebo one month later	PCV13, followed by PPSV23 one month later	PCV20 demonstrated immunogenicity across all serotypes, with immune responses somewhat lower than PCV13 for shared serotypes with no large differences observed, and generally higher than PPSV23 for additional serotypes, with a well-tolerated safety profile comparable to PCV13.
Haranaka et al., 2024 [78]	Phase 3, randomized, double-blind study	1421 adults aged ≥60 years	Participants with preexisting stable disease with an elevated risk of pneumococcal disease	Yes	PCV20, followed by a saline injection one month later	PCV13, followed by PPSV23 one month later	PCV20 induced robust immune responses across all serotypes, showing noninferior responses to PCV13 for all 13 shared serotypes and to PPSV23 for six additional serotypes, with a well-tolerated safety profile comparable to PCV13.

* Smoking, diabetes, and chronic pulmonary disease. ** Pre-existing stable disease: Individuals not requiring significant change in therapy in the previous 6 weeks or hospitalization for worsening disease within 12 weeks before receipt of the vaccine), PCV13: 13-valent pneumococcal conjugate vaccine; PCV20: 20-valent pneumococcal conjugate vaccine; PPSV23: 23-valent pneumococcal polysaccharide vaccine; QIV: Quadrivalent influenza vaccine.

**Table 4:** Expert opinions on PCV20 informed by literature evidence.

Age or Conditions	Vaccine History	Expert Opinions
18–49 Conditions: Established/guideline-based indications: Diabetes mellitus|Chronic heart disease|Chronic liver disease|Autoimmune inflammatory rheumatic diseases|Rheumatoid arthritis|SLE|Patients on DMARDs|Patients on immunosuppressive medications |Patients on chemotherapy |HIV|Immunocompromised patients|Solid organ malignancy|Hematological malignancy|Recipients of solid organ transplants |Sickle cell disease|Congenital or acquired asplenia/splenic dysfunction|Complement deficiencies|CSF leaks|Cochlear implants |CKD|Renal transplant|Hemodialysis|Chronic lung disease|COPD| Asthma|ILD|Post-TB|Emphysema |Smokers|Alcoholics. Additional groups proposed by the expert panel (not established stand-alone indications; vaccinate based on age and underlying medical risk rather than the exposure itself): Healthcare workers|People attending mass gatherings (e.g., Kumbh/Hajj/Umrah)|Environmental pollution exposure|Post-COVID sequelae| Neurological disease (e.g., multiple sclerosis) receiving immunomodulatory/immunosuppressive therapy	Vaccine-naïve/history unknown	Administer a single dose of PCV20
	PCV13 only	Administer a single dose of PCV20 after a ≥1-year interval since the last PCV13 dose
	PPSV23 only	Administer a single dose of PCV20 after a ≥1-year interval since the last PPSV23 dose
	PCV13 followed by PPSV23	High-risk: Administer one dose of PCV20 ≥ 5 years after the last pneumococcal vaccine At-risk: The pneumococcal vaccination recommendations should be reviewed again when the person turns 50 years of age Note: Review the risk at 50 years and take a shared clinical decision about PCV20 vaccination
≥50 years	Vaccine-naïve/history unknown	Administer a single dose of PCV20
	PCV13 only	Administer a single dose of PCV20 after a ≥1-year interval since the last dose of PCV13
	PPSV23 only	Administer a single dose of PCV20 after a ≥1-year interval since the last PPSV23 dose
	PCV13 followed by PPSV23	Vaccination status should be reviewed using shared clinical decision-making
		Adults aged <65 years: Administer PCV20 ≥5 years after the last pneumococcal vaccine
		Adults aged ≥65 years: After shared clinical decision-making, administer PCV20 ≥ 5 years after the last pneumococcal vaccine

CKD: Chronic kidney disease; COPD: Chronic obstructive pulmonary disease; COVID: Coronavirus disease 2019; CSF: Cerebrospinal fluid; DMARD: Disease-modifying antirheumatic drug; HIV: Human immunodeficiency virus; ILD: Interstitial lung disease; PCV13: 13-valent pneumococcal conjugate vaccine; PCV20: 20-valent pneumococcal conjugate vaccine; PPSV23: 23-valent pneumococcal polysaccharide vaccine; SLE: Systemic lupus erythematosus; TB: Tuberculosis. Note: Conditions are listed as either established, guideline-based indications for adult pneumococcal vaccination or as additional risk groups proposed by the expert panel; the latter (healthcare workers, attendance at mass gatherings, environmental pollution exposure, and post-COVID sequelae) are not recognized as stand-alone indications in major international guidelines, and vaccination in these groups should be guided by age and underlying medical risk. In patients with neurological disease such as multiple sclerosis, PCV20 should preferably be administered before initiating disease-modifying therapy; where this is not possible, timing should be individualized because such therapies may reduce vaccine immunogenicity. Recipients of hematopoietic stem cell transplants: Administer a 3+1 pneumococcal immunization schedule, consisting of a primary series of three doses of PCV20 given 3–6 months after hematopoietic stem cell transplantation (HSCT), at intervals of at least four weeks. Administer a booster (fourth) dose of PCV20 six months after completion of the primary series.

**Table 5:** Dose of prednisone and pneumococcal vaccination [109].

Dose	PCV20 Vaccination
Prednisone ≤10 mg daily or >10 mg and <20 mg daily *	Give
Prednisone ≥20 mg daily *	Defer **
PCV20: 20-valent pneumococcal conjugate vaccine.	

* Or the equivalent dose of any other glucocorticoid formulation, or the equivalent pediatric dose. ** Defer vaccination until glucocorticoids are tapered to the equivalent of prednisone <20 mg daily. PCV20: 20-valent pneumococcal conjugate vaccine.

**Table 6:** Medication management at the time of non-live attenuated vaccine administration [109].

Medication	Non-Live Vaccine (e.g., PCV20)
Methotrexate	Continue methotrexate; administer PCV20 without withholding methotrexate.
Rituximab	In patients about to start rituximab, administer PCV20 at least 2–4 weeks before the first dose. In patients already receiving rituximab, time vaccination 6 months after the last rituximab dose, and hold rituximab for at least 2 weeks after vaccination where clinically feasible.
Immunosuppressive medications other than methotrexate and rituximab	Continue non-live vaccination at the time of immunosuppressive medication, as holding medications could be associated with disease flare.

PCV20: 20-valent pneumococcal conjugate vaccine.

**Table 7:** Specialty-specific PCV20 expert opinions for pneumococcal vaccination.

Specialty/Clinical Domain	Scenarios	Expert Opinions
Pulmonology	Patients receiving corticosteroids or other immunosuppressive therapy	Tapering high-dose corticosteroids should be considered when clinically appropriate. PCV20 may be administered in patients receiving prednisone at doses of <20 mg/day, whereas vaccination should be deferred in those receiving doses of ≥20 mg/day.
	Individuals with COPD, asthma, ILDs, and post-TB lung disease	PCV20 is advised due to the increased risk of pneumococcal disease.
Nephrology	All CKD stages and nephrotic syndrome	PCV20 should be initiated early to reduce the burden and risk of pneumococcal disease.
	Dialysis	Pneumococcal vaccination is recommended prior to the initiation of dialysis.
Endocrinology	Glycemic status (DM)	PCV20 can be administered irrespective of glycemic status. The optimal window for pneumococcal immunization is within the first six months after diagnosis of diabetes.
Cardiology	Heart disease	PCV20 immunization is advised for adults with cardiac conditions to reduce the risk of pneumococcal disease and associated sequelae.
Rheumatology	Patients with rheumatic and musculoskeletal diseases receiving immunosuppressive therapy	PCV20 should be administered before starting immunosuppressive therapy. In patients starting rituximab, PCV20 should be given at least 2–4 weeks before the first dose; in patients already receiving rituximab, time vaccination 6 months after the last rituximab dose, and hold rituximab for at least 2 weeks after vaccination where feasible. PCV20 may be administered during methotrexate therapy or other ongoing immunosuppressive treatments. PCV20 may be administered in patients receiving prednisone at doses <20 mg/day; however, in those receiving ≥20 mg/day, vaccination should be deferred.
Hematology	Thrombocytopenia or coagulation disorders	Clinicians should carefully assess bleeding risk before IM injections and consider SC administration when the clinical benefit outweighs potential risks. Vaccination should ideally be scheduled shortly after hemostatic treatment, when applicable.
	HSCT	PCV20 should be administered at least two weeks before transplantation, when feasible, or starting at three months after HSCT as a three-dose series at four-week intervals, followed by a booster dose six months after the primary vaccination series.
Oncology	Chemotherapy in hematological malignancies	PCV20 should ideally be administered 2–4 weeks before initiation of chemotherapy. PCV20 may also be given during treatment or at least three months after completion of chemotherapy.
	Chemotherapy in solid organ malignancies	PCV20 is recommended at the time of cancer diagnosis and prior to initiation of chemotherapy or radiotherapy to ensure an optimal immune response.
Solid organ transplant	Heart, kidney, liver	PCV20 should preferably be administered at least 2 weeks before transplantation or, if deferred, 3–6 months after transplantation, as a single dose.

CKD: Chronic kidney disease; COPD: Chronic obstructive pulmonary disease; DM: Diabetes mellitus; HSCT: Hematopoietic stem cell transplantation; ILD: Interstitial lung disease; IM: Intramuscular; PCV20: 20-valent pneumococcal conjugate vaccine; SC: Subcutaneous; TB: Tuberculosis.

## Data Availability

No new data were created or analyzed in this study. Data sharing is not applicable to this article.

## Supplementary Materials

The following supporting information can be downloaded at https://www.mdpi.com/article/10.3390/vaccines14100905/s1: Table S1: Evidence basis for the principal PCV20 expert opinions; Table S2: Current national and international recommendations for the use of PCV20 [11,17,92,93,94,95,96,97,99,100]; Figure S1: Expert recommendations for PCV20 in patient profiles.

## Abbreviations

The following abbreviations are used in this manuscript:

ACIP	Advisory Committee on Immunization Practices
ACP	All-cause pneumonia
AMR	Antimicrobial resistance
CDC	Centers for Disease Control and Prevention
CKD	Chronic kidney disease
COPD	Chronic obstructive pulmonary disease
CAP	Community-acquired pneumonia
CAPiTA	Community-Acquired Pneumonia Immunization Trial in Adults
GINA	Global Initiative for Asthma
GOLD	Global Initiative for Chronic Obstructive Lung Disease
HCP	Healthcare professional
HSCT	Hematopoietic stem cell transplantation
ICU	Intensive care unit
ILD	Interstitial lung disease
ISPOR	International Society for Pharmacoeconomics and Outcomes Research
IPD	Invasive pneumococcal disease
NCCN	National Comprehensive Cancer Network
NGT	Nominal Group Technique
PGD	Patient Group Direction
PCV	Pneumococcal conjugate vaccine
PCV13	13-valent pneumococcal conjugate vaccine
PCV20	20-valent pneumococcal conjugate vaccine
PPSV23	23-valent pneumococcal polysaccharide vaccine
PTLD	Post-tuberculosis lung disease
QALY	Quality-adjusted life year
RMD	Rheumatic and musculoskeletal disease
SHARE	Share, Highlight, Address, Remind, and Explain
*S. pneumoniae*	*Streptococcus pneumoniae*
UK	United Kingdom
US	United States
VT	Vaccine-type
VT-CAP	Vaccine-type community-acquired pneumonia
WHO	World Health Organization
