# Supplementary material for: Indian Multidisciplinary Expert Opinions on the Use of 20-Valent Pneumococcal Conjugate Vaccine in Adults
Source: Vaccines (Basel). 2026 Oct 16;14(10):905. doi: 10.3390/vaccines14100905 (PMC13709873; doi:10.3390/vaccines14100905)
Supplement: Supplementary file 1 [file vaccines-14-00905-s001.zip › vaccines-4546895-supplementary.pdf]

Supplementary Materials

Supplementary Tables

Supplementary Table S1: Evidence basis for the principal PCV20 expert opinions

Major Expert opinions	Predominant basis of evidence	Nature of supporting data
Single PCV20 dose for all adults aged ≥50 years	Direct PCV20 data; guideline extrapolation	Phase 3 immunogenicity/safety data for PCV20; alignment with international and national adult schedules
Single PCV20 dose for adults aged 18–49 years with at-risk/high-risk conditions	Guideline extrapolation; expert opinion	Extrapolated from risk-based pneumococcal recommendations; panel consensus for the Indian context
PCV20 preferred over sequential PCV13→PPSV23 (schedule simplification)	Direct PCV20 data; expert opinion	Single-dose broad coverage; panel view on improved feasibility and adherence
Expanded serotype coverage addresses residual IPD/CAP not covered by PCV13	Indirect data	Serotype-distribution and coverage studies from Indian and global surveillance
Retention of a ≥1-year interval before PCV20 in prior PCV13 recipients	Guideline extrapolation; expert opinion	Based on existing interval guidance; panel recommends retaining ≥1 year rather than shortening it
PCV20 is cost-saving/cost-effective	Indirect data (economic modelling)	UK and Argentina cost-effectiveness models and preliminary Indian analysis (conference data; transferability to India uncertain)
PCV20 may help mitigate antimicrobial resistance (AMR)	Indirect data; expert opinion	Serotype–antimicrobial-resistance associations; available Indian datasets are small and lack serotype-level data, so this is interpreted cautiously
Vaccination in specific risk conditions (renal disease/dialysis/transplant; rheumatologic/immunosuppressed patients; asplenia; cochlear implants; CSF leak; neurological disease)	Guideline extrapolation; expert opinion	Established risk-group indications complemented by panel guidance for Indian practice
Timing of PCV20 around immunosuppressive/B-cell-depleting therapy (e.g., rituximab; before initiating disease-modifying therapy)	Guideline extrapolation; expert opinion	General vaccinology principles on immunogenicity; specific timing specified by the panel

Additional panel-proposed groups (healthcare workers, mass gatherings, environmental pollution, post-COVID sequelae)	Expert opinion	Not established stand-alone indications; contextual suggestions of the expert panel

AMR: antimicrobial resistance; CAP: community-acquired pneumonia; CSF: cerebrospinal fluid; IPD, invasive pneumococcal disease; PCV13/PCV20, 13-/20-valent pneumococcal conjugate vaccine; PPSV23, 23-valent pneumococcal polysaccharide vaccine.

Supplementary Table S2: Current national and international recommendations for the use of PCV20

Guidelines	Recommendations
National Guidelines – India	
Indian Consensus Guideline on Adult Immunization [11]	PCV20 are recommended for adults<br>• Above 50 years: 1 dose of PCV20 only, OR PCV15* followed by PPSV23 1 year later<br>• At-risk: 1 dose of PCV20 only, OR PCV15* followed by PPSV23 1 year later<br>• High-risk: 1 dose of PCV20 only, OR PCV15* followed by PPSV23 8 weeks later
International Guidelines	
CDC ACIP [17]	Adults aged ≥50 years:<br>• None or PCV7 only at any age: 1 dose of PCV15, PCV20, or PCV21. Additional vaccination of PPSV23 is not indicated in case PCV20 or PCV21 is administered.<br>• Previously received only PCV13: 1 dose of PCV20 or PCV21 ≥1 year after the last PCV13 dose.<br>• Previously received only PPSV23: 1 dose of PCV15, PCV20, or PCV21 ≥1 year after the last PPSV23 dose.<br>• PCV13 at any age and PPSV23 at age <65 years: 1 dose of PCV20 or PCV21 ≥5 years after the last pneumococcal vaccine dose.<br>• PCV13 at any age and PPSV23 at age ≥65 years: Shared clinical decision-making is recommended regarding administration of either a single dose of PCV20 or PCV21. If a decision to administer PCV20 or PCV21 is made, a single dose is recommended ≥5 years after the last pneumococcal vaccine dose.<br><br>Adults aged 19–49 years with an immunocompromising condition,† a CSF leak, or a cochlear implant:<br>• None or PCV7 only at any age: 1 dose of PCV15, PCV20, or PCV21. If PCV15 is used, administer a single dose of PPSV23* ≥8 weeks after the PCV15 dose.<br>• Previously received only PCV13: 1 dose of PCV20 or PCV21 administered ≥1 year after the PCV13 dose.

Guidelines	Recommendations
	• Previously received only PPSV23: 1 dose of PCV15, PCV20, or PCV21 ≥1 year after the last PPSV23 dose.<br>• PCV13 and 1 dose of PPSV23: 1 dose of PCV20 or PCV21 ≥5 years after the last pneumococcal vaccine dose. The pneumococcal vaccination series is complete, and it need not be followed by additional pneumococcal vaccine doses.<br>• PCV13 and two doses of PPSV23: The pneumococcal vaccination recommendations should be reviewed again when the person turns 50 years old. Alternatively, a single dose of either PCV20 or PCV21 should be administered ≥5 years after the last pneumococcal vaccine dose. *If PCV20 or PCV21 is used, the series is complete, and it need not be followed by additional pneumococcal vaccine doses.*<br>Adults aged 19–49 years with chronic medical conditions§:<br>• None or PCV7 only at any age: 1 dose of PCV15, PCV20 or PCV21. If PCV15 is administered, a single dose of PPSV23* should be administered ≥1 year after the PCV15 dose.<br>• PCV13 only: 1 dose of PCV20 or PCV21 ≥1 year after the PCV13 dose.<br>• PPSV23 only: 1 dose of PCV15, PCV20, or PCV21 ≥1 year after the last PPSV23 dose.<br>• PCV13 and 1 dose of PPSV23: The pneumococcal vaccination recommendations should be reviewed again when the person reaches age 50 years.<br>*For adults who have received PCV15 but have not completed their recommended pneumococcal vaccine series with PPSV23, 1 dose of PCV21 or PCV20 may be used if PPSV23 is not available.<br>†Chronic renal failure, nephrotic syndrome, immunodeficiency, iatrogenic immunosuppression, generalized malignancy, HIV infection, Hodgkin disease, leukemia, lymphoma, multiple myeloma, solid organ transplant, congenital or acquired asplenia, or sickle cell disease or other hemoglobinopathies.<br>§Alcoholism; chronic heart disease, including congestive heart failure and cardiomyopathies; chronic liver disease; chronic lung disease, including chronic obstructive pulmonary disease, emphysema, and asthma; cigarette smoking; or diabetes mellitus.
American Academy of Family Physicians (AAFP) [92]	**Same as CDC ACIP recommendations**
American Diabetes Association [97]	• Adults aged ≥65 years whose vaccine status is unknown or who have not received pneumococcal vaccine: One dose of PCV15 or PCV20. If PCV15 is used, it should be followed by PPSV23.

Guidelines	Recommendations
	• Adults aged 19–64 years with certain underlying risk factors or other medical conditions whose vaccine status is unknown or who have not received pneumococcal vaccine: One dose of PCV20 or PCV15<br>• If PPSV23 is the only dose received, PCV15 or PCV20 may be given ≥1 year later.
National Advisory Committee on Immunization (NACI) [93]	Adults (≥18 years) without IPD risk factors:<br>• One dose of PCV20 or PCV21 should be offered to all adults 65 years of age, regardless of their pneumococcal vaccination status with PCV13, PCV15, or PPSV23.<br>• In previously immunized adults 65 years of age and older, PCV20 or PCV21 should be provided at least 1 year from either the last dose of PCV13, PCV15, or PPSV23.<br>Adults (≥18 years) with IPD risk factors:<br>• Regardless of their pneumococcal vaccination history with PCV13, PCV15 or PPSV23, one dose of PCV20 or PCV21 is recommended for adults with IPD risk factors.
Australian Technical Advisory Group on Immunization [99]	Adults:<br>• A single dose of PCV13, PCV15, or PCV20 is recommended for all non-Indigenous adults at 70 years of age.<br>• Non-Indigenous adults aged ≥70 years who did not receive a dose of a pneumococcal conjugate vaccine at 70 years of age are recommended to receive a single catch-up dose of PCV13, PCV15, or PCV20 as soon as possible.<br>• Non-Indigenous adults aged ≥70 years who have previously received PPSV23 and have not already received a conjugate vaccine are recommended to receive a single dose of PCV13, PCV15, or PCV20 at least 12 months after their last PPSV23 dose.<br>Aboriginal and Torres Strait Islander adults without risk conditions for pneumococcal disease are recommended to receive:<br>• a dose of a pneumococcal conjugate vaccine (PCV13, PCV15 or PCV20) at age ≥50 years, and<br>• a dose of PPSV23 12 months later, and<br>• a 2nd dose of PPSV23 at least 5 years later<br>Aboriginal and Torres Strait Islander adults with risk conditions for pneumococcal disease are recommended to receive one dose of a conjugate vaccine (PCV13, PCV15, or PCV20) at diagnosis, followed by two doses of PPSV23.

Guidelines	Recommendations
**German Standing Committee on Vaccination (STIKO)** [100]	• For ≥ 18 years of age who have already received a sequential vaccination (PCV13/PCV15 + PPSV23) or a single PPSV23 vaccination (Group 2: Other chronic diseases) in the past should receive a PCV20 at least 6 years after the PPSV23 vaccination<br>• In the case of severe immunodeficiency, vaccination with PCV20 can be administered at least 1 year after the PPSV23 vaccination.<br>• For age ≥60 years who have been vaccinated with the PPSV23 in the past should receive a vaccination with PCV20 at an interval of 6 years after PPSV23 vaccination<br>• For occupational activities (such as welding and cutting of metals that result in exposure to metal fumes, including metal oxide welding fumes, and smoke) who have been vaccinated with the PPSV23 in the past should receive a vaccination with PCV20 at an interval of 6 years after PPSV23 vaccination.
European Centre for Disease Prevention and Control (ECDC) [94]	• Belgium: Preferred scheme: one of PCV20 for alternative schemes based on past pneumococcal vaccination.<br>• France: From August 2023, PCV20 recommended instead of a mixed schedule PCV13 + PPSV23 in adults from 18 years of age for individuals at risk.<br>• Germany: For individuals previously vaccinated with PPSV23, a dose of PCV20 is recommended in an interval of at least 6 years after the PPSV23 dose.<br>• Greece: PCV20 is recommended in ≥50 years age.<br>• Hungary: PCV20 is recommended as catch-up vaccination in ≥60 years age.<br>• Iceland: One dose of polysaccharide vaccine or PCV20 if the polysaccharide vaccine is not available or according to medical advice.
National Comprehensive Cancer Network (NCCN) [95]	• The PCV20 should be administered to newly diagnosed adults with cancer who are pneumococcal vaccine naïve.<br>• For patients who have previously received PPSV23, PCV20 (preferred) or PCV15 can be given.<br>• Patients who have previously received PCV13 only can receive PCV20 (rather than PPSV23) at least 1 year later.<br>• Patients who have previously received PCV13 and 1 to 2 doses of PPSV23 can receive PCV20 at least 5 years later.

Guidelines	Recommendations
Kamboj, M., et al 2024 [96]	• One dose of PCV20 only or a dose of PCV15 followed by a dose of PPSV23 with at least 8 weeks gap between 2 administrations is recommended in adults ≥19 years with cancer.<br>• The current US recommendation is to revaccinate all HSCT recipients with the first dose of PCV20 at 4-6 months after transplant since this adds coverage for additional pneumococcal serotypes. The subsequent two doses are given at 1-month intervals, followed by the fourth dose administered 6 months later.<br>• Patients who have started revaccination with PCV15 can complete subsequent doses with PCV20.

ACIP: Advisory Committee on Immunization Practices; CDC: Centers for Disease Control and Prevention; PCV13: 13-valent pneumococcal conjugate vaccine; PCV15: 15-valent pneumococcal conjugate vaccine; PCV20: 20-valent pneumococcal conjugate vaccine; PPSV23: 23-valent pneumococcal polysaccharide vaccine.

Supplementary Figure Legends

Color code				
**Recommendations**	Administer a single dose of PCV20	Administer a single dose of PCV20 after a ≥1-year interval since the last PCV13 or PPSV23 dose	High-risk: Administer 1 dose of PCV20 ≥5 years after the last pneumococcal vaccine At-risk: The pneumococcal vaccination recommendations should be reviewed again when the person turns age 50	Administer PCV20 ≥5 years after the last pneumococcal vaccine (aged <65 years) PCV13 at any age and PPSV23 at age ≥65 years: Shared clinical decision-making to give PCV20 or not

Patient profile	Expert recommendation							
	Age: 18–49 (co-morbidities)				Age: ≥50 years			
	Vaccine Naïve/ History unknown	PCV13 only	PPSV23 only	PCV13 f/b PPSV23	Vaccine Naïve/ History unknown	PCV13 only	PPSV23 only	PCV13 f/b PPSV23
Diabetes								
Chronic heart disease								
Autoimmune inflammatory rheumatic diseases*								
Rheumatoid arthritis*								
SLE*								
Patients on DMARDs*								
Patients on immunosuppressive medication								
Patients on chemotherapy**								
HIV								
Immunocompromised								
Patients with solid organ malignancy***								
Patients with hematological malignancy#								
Recipients of solid organ transplants								
Patients with sickle cell disease								
Compliment deficiencies								
CSF leaks								
Chronic kidney disease^								
Renal transplant								
Hemodialysis								
Healthcare worker								
Travelers								
Mass gatherings (Kumbh/Hajj/Umrah)								
Chronic lung disease								
COPD								
Asthma								
ILD@								
Post-Tb@								
Emphysema								
Smokers								
Alcoholics								
Immunosuppressive medications								
Environmental pollution								
Post-COVID sequelae@								

*The CDC ACIP 2024 recommendations define the range of conditions considered immunocompromising. The recommendations provided here are based on classifying this condition as immunocompromised or immunosuppressed, using available evidence related to the disease itself and its management. The condition is associated with immunosuppression, either due to the underlying autoimmune disorder or the immunosuppressive therapies used. Therefore, the vaccination schedule applied is recommended for individuals with immunosuppression.

**The recommendations provided are based on those outlined for patients with cancer in the CDC ACIP 2024 guidelines.

***The recommendations provided are based on CDC ACIP 2024 recommendations for generalized malignancy, as the guidelines do not specifically distinguish solid organ malignancies.

#The recommendations provided are based on CDC ACIP 2024 recommendations for leukemia, lymphoma, and multiple myeloma, as the guidelines do not explicitly mention hematologic malignancies.

^The recommendations provided are according to the CDC ACIP 2024 guidelines for renal transplant and hemodialysis.

@The recommendations provided are according to the CDC ACIP 2024 guidelines for chronic lung diseases, including chronic obstructive pulmonary disease, emphysema, and asthma.

Note: Recipients of hematopoietic stem cell transplants: Administer a 3+1 pneumococcal immunization schedule, consisting of a primary series of three doses of PCV20 given 3–6 months after HSCT, at intervals of at least 4 weeks. Administer a booster (fourth) dose of PCV20 six months after completion of the primary series.

COPD: Chronic obstructive pulmonary disease; **CSF:** Cerebrospinal fluid; **DMARD:** Disease-modifying anti-rheumatic drug; **HIV:** Human immunodeficiency virus; HSCT: Hematopoietic stem cell transplantation;**ILD:** Interstitial lung disease; **PCV13:**13-valent pneumococcal conjugate vaccine; **PCV20:** 20-valent pneumococcal conjugate vaccine; **PPSV23:** 23-valent pneumococcal polysaccharide vaccine; **Post-COVID:** Post-coronavirus disease sequelae; **Post-Tb:** Post-tuberculosis; **SLE:** Systemic lupus erythematosus.

Supplementary Figure S1: Expert recommendations for PCV20 in patient profiles

COPD: Chronic obstructive pulmonary disease; CSF: Cerebrospinal fluid; DMARDs: Disease-modifying anti-rheumatic drugs; HIV: Human immunodeficiency virus; ILD: Interstitial lung disease; PCV13: 13-valent pneumococcal conjugate vaccine; PCV20: 20-valent pneumococcal conjugate vaccine; PPSV23: 23-valent pneumococcal polysaccharide vaccine; Post-COVID: Post-corona-virus disease sequelae; Post-TB: Post-tuberculosis; SLE: Systemic lupus erythematosus.

[*] *The CDC ACIP 2024 recommendations define the range of conditions considered immunocompromising. The recommendations provided here are based on classifying this condition as immunocompromised or immunosuppressed, using available evidence related to the disease itself and its management. The condition is associated with immunosuppression, either due to the underlying autoimmune disorder or the immunosuppressive therapies used. Therefore, the vaccination schedule applied is that recommended for individuals with immunosuppression.*

[**] *The recommendations provided are based on those outlined for patients with cancer in the CDC ACIP 2024 guidelines.*

[***] *The recommendations are provided based on CDC ACIP 2024 recommendations for generalized malignancy, as the guidelines do not specifically distinguish solid organ malignancies.*

[#] *The recommendations are provided based on CDC ACIP 2024 recommendations for leukemia, lymphoma, and multiple myeloma, as the guidelines do not explicitly mention hematologic malignancy.*

[$] *The CDC ACIP 2024 recommendations do not include guidance for hematopoietic stem cell transplant. Therefore, the recommendations for hematopoietic stem cell transplant have been added based on the CDC ACIP 2023 guidelines.*

[^] *The recommendations are provided according to the CDC ACIP 2024 guidelines for renal transplant and hemodialysis*

[@] *The recommendations are provided according to the CDC ACIP 2024 guidelines for chronic lung diseases, including chronic obstructive pulmonary disease, emphysema, and asthma.*

Note: Conditions are listed as either established, guideline-based indications for adult pneumococcal vaccination or as additional risk groups proposed by the expert panel; the latter (healthcare workers, attendance at mass gatherings, environmental pollution exposure, and post-COVID sequelae) are not recognized as stand-alone indications in major international guidelines, and vaccination in these groups should be guided by age and underlying medical risk. In patients with neurological disease such as multiple sclerosis, PCV20 should preferably be administered before initiating disease-modifying therapy; where this is not possible, timing should be individualized because such therapies may reduce vaccine immunogenicity.
